# Spatial heterogeneity of malaria in Ghana: a cross-sectional study on the association between urbanicity and the acquisition of immunity

**DOI:** 10.1186/s12936-016-1138-4

**Published:** 2016-02-11

**Authors:** Clemens Frank, Ralf Krumkamp, Nimako Sarpong, Peter Sothmann, Julius N Fobil, Geoffrey Foli, Anna Jaeger, Lutz Ehlkes, Ellis Owusu-Dabo, Yaw Adu-Sarkodie, Florian Marks, Ralf R. Schumann, Jürgen May, Benno Kreuels

**Affiliations:** Research Group Infectious Disease Epidemiology, Bernhard Nocht Institute for Tropical Medicine, Hamburg, Germany; Institute of Microbiology and Hygiene, Charité-University Medicine, Berlin, Germany; German Centre for Infection Research (DZIF), Partner Site Hamburg-Borstel-Lübeck, Hamburg, Germany; Kumasi Centre for Collaborative Research in Tropical Medicine, College of Health Sciences, KNUST, Kumasi, Ghana; Division of Tropical Medicine, 1st Department of Medicine, University Medical Centre Hamburg Eppendorf, Hamburg, Germany; School of Public Health, University of Ghana, Accra, Ghana; School of Medical Sciences, Kwame Nkrumah University of Science and Technology, Kumasi, Ghana; International Vaccine Institute, Seoul, Korea

**Keywords:** Malaria, Ghana, Urbanization, Semi-immunity, Children

## Abstract

**Background:**

Malaria incidence has declined considerably over the last decade. This is partly due to a scale-up of control measures but is also attributed to increasing urbanization. This study aimed to analyse the association between malaria and urbanization and the effect of urbanicity on the acquisition of semi-immunity.

**Methods:**

In 2012, children with fever presenting to St Michael’s Hospital Pramso/Ghana were recruited. The malaria-positive-fraction (MPF) of fever cases was calculated on community-level to approximate the malaria risk. The mean age of malaria cases was calculated for each community to estimate the acquisition of semi-immunity. The level of urbanicity for the communities was calculated and associations between MPF, urbanicity and immunity were modelled using linear regression.

**Results:**

Twenty-six villages were included into the study with a mean MPF of 35 %. A linear decrease of 5 % (95 % CI: 4–6 %) in MPF with every ten-point increase in urbanicity was identified. The mean age of malaria patients increased by 2.9 months (95 % CI: 1.0–4.8) with every ten-point increase in urbanicity.

**Discussion:**

The results confirm an association between an increase in urbanicity and declining malaria risk and demonstrate that the acquisition of semi-immunity is heterogeneous on a micro-epidemiological scale and is associated with urbanicity.

**Electronic supplementary material:**

The online version of this article (doi:10.1186/s12936-016-1138-4) contains supplementary material, which is available to authorized users.

## Background

*Plasmodium falciparum* malaria is the most important parasitic disease worldwide with an estimated 1.1–1.4 billion people at risk of infection [1]. Despite a strong decrease in incidence and mortality in the last decade, the World Health Organization (WHO) estimates a total of 207 million clinical cases of malaria, resulting in approximately 627,000 deaths in 2012 [2]. The recent decline in incidence is largely due to a huge scale-up of malaria control efforts since 2003. However, the decline started before efforts were intensified and has occurred in regions that have not benefitted from these measures [3]. Among others, this effect has been attributed to economic development and urbanization in endemic areas [4]. The process of urbanization, including landscape modification, transformation of the environment and economic change, leads to entomological, parasitological and human behavioural changes reducing malaria transmission intensity [3, 5]. It is estimated that currently one-third of the world’s population lives in urban areas and further growth of urban populations is expected. This increase will mainly occur in the less developed regions of the world with a predicted rise from 0.41 billion in 2011 to 1.26 billion urban inhabitants in 2050 in the African region [6]. The speed of this process is particularly dramatic in West Africa, with an urban population growth rate of 6.3 % per annum [7].

Earlier studies on the differences between urban and rural malaria transmission often made comparisons on a regional [8] or even global scale [1, 3, 9]. Most of these studies compared malaria transmission on a binary scale between urban areas and a disjoint rural region, sometimes separated by hundreds of kilometres. However, this approach does not capture malaria transmission adequately as spatial heterogeneity is already observed over short distances [10], even between households [11].

It has been shown that changes in transmission intensity also influence the acquisition of immunity against malaria [12]. With declining transmission, the average age of patients with malaria increases and severe malaria also occurs in older children and adults. It is unclear to what extent urbanization influences the acquisition of immunity and how immunity varies over short distances. Understanding the heterogeneity of transmission on a micro-epidemiological scale and how it influences the acquisition of immunity is essential for an effective application of control measures and the development of new intervention strategies [11, 13, 14].

The aim of this study was to study the association between urbanicity and malaria and how this may influence the development of immunity on a micro-epidemiological level.

## Methods

The study was conducted in the Bosomtwe district of the Ashanti Region in Ghana. Two sets of data were used for the analysis: (1) an individual-level survey containing data on residence, disease status and demography of children attending a local hospital; and, (2) a community-level survey containing data on community characteristics of villages located within the hospital catchment area.

### Study site and study area

The Ashanti Region is located in the forest-belt of Ghana, characterized by wet tropical climate with a main rainy season from March to July and a minor rainy season from September to November. The vegetation in the study area is dominated by semi-deciduous forest, but farming activities thinned out much of the former closed forests. Malaria is endemic with high transmission intensity throughout the year [15].

### Hospital survey

Patients were recruited at St Michael’s Hospital (SMH) in Pramso, a town of about 3300 inhabitants located 20 km southeast of the regional capital Kumasi, on one of the major roads leaving the city. SMH is the biggest health facility in the Bosomtwe district with a large general outpatient department (OPD) and four wards, one each for women, children and men and a maternity ward. The catchment area of SMH stretches from urban Kumasi and its suburbs in the northwest to rural areas of the Bosumtwe District in the southeast.

All children <15 years of age with fever (tympanic temperature ≥38 °C) presenting to the OPD during the study period (January to December 2012) were recruited if informed consent was given by their caregiver. A blood sample was taken by venipuncture and a questionnaire on basic demographic data was completed. Children were treated according to hospital guidelines. Children attending the hospital for a second time were only considered as a new case if this visit occurred >28 days after a previous visit.

### Laboratory diagnostics

Thick and thin blood films for malaria parasite examination were prepared according to quality-controlled, standardized protocols. Two independent readers examined the slides and the mean of these readings was used to quantify parasitaemia as asexual parasites/µl. A third reading was performed in case of discrepancies in positivity, malaria species, or a less than threefold difference in quantification. An episode of malaria was defined as fever accompanied by an asexual parasite count of ≥5000/µl.

### Community data

**Fig. 1:**
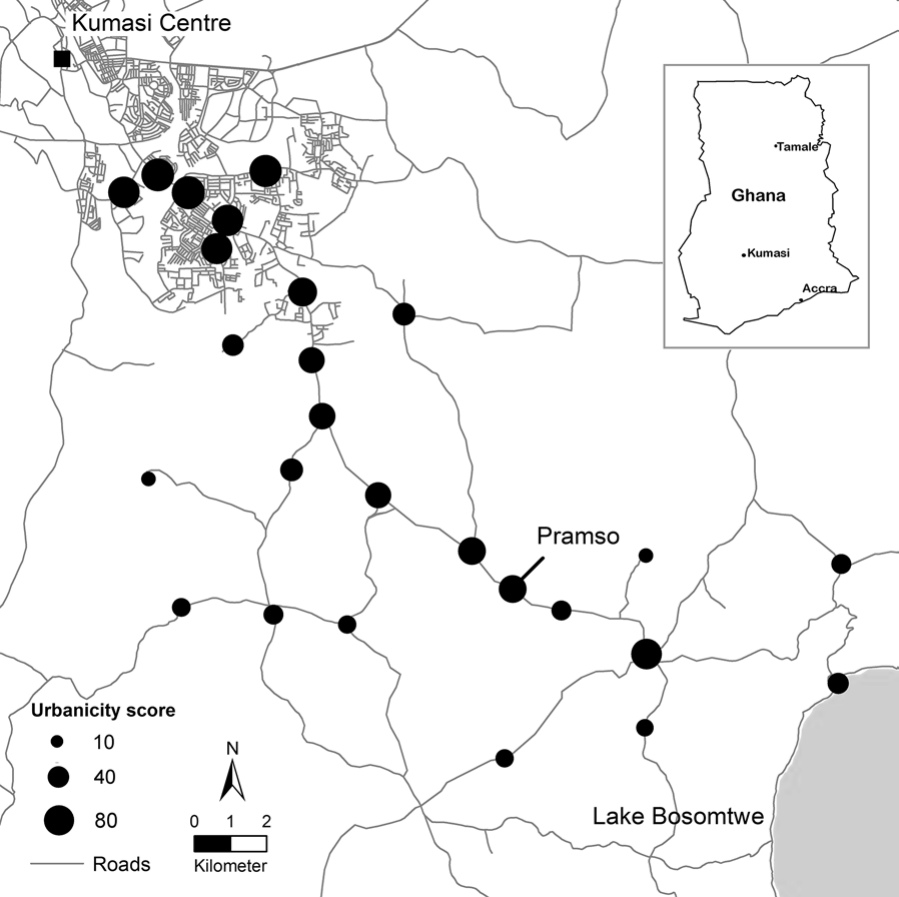
Map of the study area. *Black circles* represent study communities

The catchment area of SMH was defined as a geographically continuous area around the hospital, which covered the residence of 95 % of SMH’s patients (Fig. 1). Data on community characteristics used to construct the urbanicity scale were retrieved from the ‘Ghana population and housing Census 2010’ [16]. Additional data on the availability of private services (e.g., banks, petrol stations) and information about road conditions and frequency of public transport were collected via systematic interviews conducted in each community.

### Urbanicity scale

The urbanicity scale was constructed and validated according to the principles of scale development as described by DeVellis [17] and Netemeyer and Bearden [18]. Data used to establish the scale were selected by literature review and expert opinion. Eight components were defined within which variables were grouped: population, economic activity, education, healthcare, transportation, service-infrastructure, sanitation, and housing. Each component accounted for ten points. Consequently, the final scale ranged from 0 to 80 points. The components used to construct the scale are displayed in Table 1. To calculate each community’s urbanicity, score points were accumulated according to its particular characteristics. Unidimensionality of the scale was tested by exploratory factor analysis, Kaiser–Meyer–Olkin measure was used to assess sampling adequacy and internal consistency was validated using Cronbach’s alpha. For more details on scale construction and validation see Additional files 1 and 2.

**Table 1:** Algorithm used to assess the level of urbanicity

Components	Variables	Score-points	Variation
Population size	Population	0–10	214–72,105
Economic activity	Proportion of employed people in the non-agricultural sector	0–10	11.5–98.1 %
Education	Availability of primary school	0/2	
	Availability of junior high school	0/2	
	Availability of senior high school	0/2	
	Proportion of literate people	0–2 (0.5)	61.2–93.3 %
	Proportion of people with postgraduate education	0–2 (0.5)	0.5–18.9 %
Health services	Availability of a clinic^a^	0/5	
	Availability of a hospital^a^	0/5	
Transportation	Road condition^a^	0/5	
	Frequency of public transport^a^	0/5	
Services	Availability of post office^a^	0/2.5	
	Availability of petrol station^a^	0/2.5	
	Availability of bank^a^	0/2.5	
	Proportion of households connected to waste collection system	0–2.5 (1.25)	0–39 %
Sanitation	Proportion of households with tap water	0–5 (2.5)	0–64.7 %
	Proportion of households with a flush toilet	0–5 (1.25)	0–70.9 %
Housing	Proportion of households with electric light	0–2 (0.5)	37.2–96.6 %
	Proportion of households with a concrete roof	0–2 (1)	0–9.8 %
	Proportion of households with concrete walls	0–2 (0.5)	2.3–98.4 %
	Proportion of households with a concrete floor	0–2 (0.5)	1.9–100 %
	Proportion of rented dwellings	0–2 (0.5)	0–70.1 %

Linear variables were categorized and received score points depending on their quantiles, graduation in brackets

^a^Data collected via systematic interviews by the study team in 2012, other variables were retrieved from the Ghana statistical service and were collected during the 2010 National Census

### Statistical analysis

The proportion of fever cases that fulfilled the malaria criteria was calculated for each community. To retrieve robust estimates, the malaria positive fraction (MPF), i.e., number of patients with malaria over all patients with fever from each community) was calculated only for communities with at least 20 study participants. As it can be expected that in areas of low transmission older children are still susceptible to malaria, therefore leading to a higher mean age of malaria patients compared to areas of high transmission, the mean age of malaria cases in each community was calculated to approximate the local age at infection and to estimate the acquisition of immunity. Summary statistics for the study participants are presented as proportions for dichotomous or categorical variables and as medians with interquartile range (IQR) or means with standard deviation (SD) for continuous variables.

First, bivariate linear regression analysis was performed: (1) between urbanicity (score) and MPF, to assess the association between urbanicity and malaria prevalence; (2) between urbanicity (score) and the mean age of malaria patients in each community to assess an association between urbanicity and acquisition of immunity to malaria; and, (3) between MPF and mean age of malaria patients in each community to assess an association between malaria prevalence and acquisition of immunity to malaria. Second, it was decided a priori to repeat the three models including insecticide-treated nets (ITNs) as a covariate, as the use of ITNs is known to be associated with malaria as well as urbanicity [19], and could be a potential confounder.

To exclude age of patients at presentation as a potential confounder, linear regression analysis was also conducted for MPF and urbanicity and the mean age of presentation for all children from a village, irrespective of malaria positivity. As no association was seen between mean age of all patients and MPF or urbanicity, age was not included in the multivariate regression model between MPF and urbanicity. In addition, linear regression models were applied to assess the association between the particular score-components (each ranging from 0 to 10 points) and MPF to analyse which components have the strongest association with MPF. A multivariate regression model was established to assess co-linearity between variables.

All regression estimates were weighted by the number of observations from each community as the models used proportions (MPF) and means (age) as outcome measures. All measures of increase/decrease in the dependent variables were calculated based on a ten-point increase in the urbanicity score. An increase of ten points on the score would be achieved, e.g., by an increase in the proportion of households with tap water from the lowest to the highest quartile or an increase of the population from 800 to 8000 plus the availability of a post office and a bank (Table 1). All analyses were carried out using Stata v12.1 (StataCorp LP, College Station, USA).

### Ethical approval

The Committee on Human Research, Publications and Ethics, School of Medical Sciences, Kwame Nkrumah University of Science and Technology, Kumasi, Ghana approved the study design and the informed consent procedure. Participants were recruited to the study after the aims of the study and the risks and benefits were explained and the informed consent form was signed by their caregiver.

## Results

### Study participants in the hospital survey

In total, 2230 children from the catchment area attended the OPD at SMH 2594 times during the study period. The vast majority of children visited the hospital once (1942; 74.9 %) or twice (257; 9.9 %) with a maximum of five visits per individual. To exclude counting a single infection twice, all children returning ≤28 days after a previous visit were excluded from the analysis. The analysis presented here includes 2203 (84.9 %) of the visits, made by 1907 children who lived in 26 communities (≥20 observations per community) within the catchment area. A total of 1023 (46.5 %) visits were made by females and the mean age was 3.3 years (SD: ±3.2). The mothers of 1769 cases (81.9 %) stated that their child had slept under an ITN in the previous night. Table 2 summarizes the characteristics of the cases and gives an overview on the variation by community.

**Table 2:** Summary of case characteristics, overall and variation by community

Characteristics	Overall	Variation by community	
		Median	IQR
Cases, *n*	2203	49	28–101
Mean age, *years* (*SD*)	3.3 (3.2)	3.2	2.9–3.6
Female, *n* (*%*)	1023 (46.5)	24 (46.2)	13–45 (41.9–51.4)
ITN use^a^, n (%)	1769 (81.9)	43 (86.7)	24–70 (81.3–91.5)

^a^43 Missing values

### Malaria cases

During the study period, a total of 708 malaria episodes in 660 children were observed, corresponding to 32.1 % of all hospital visits. Most children (n = 620; 87.6 %) had only one malaria episode, while one child presented with malaria four times during the study period. Patients with malaria had a mean age of 3.9 years (SD: ±3.1) compared to 3 years (SD: ±3.1) in children without malaria.

### Community characteristics and urbanicity scale

Of the 26 communities included in this analysis, 20 were located in the Bosomtwe District and six were suburbs within the Kumasi Metropolitan Area. The population size of these communities ranged from 650 to 65,225 inhabitants, with a median size of 3195 (IQR: 1288–7474). The number of patients that attended SMH per study-community ranged from 21 to 369 and the median number of patients per study-community was 146 (IQR: 61–289).

The minimum score of a study-community on the urbanicity scale was 13.75 and the maximum was 77.5 points, with a median of 41 (IQR: 27–65.25) points. Validation of the scale showed good unidimensionality (eigenvalue of the first factor: 6.06 with 96.6 % of the overall variance explained), good sampling adequacy (Kaiser–Meyer–Olkin measure of 0.93 for the first factor) and a high internal consistency (Cronbach’s alpha of 0.96). The urbanicity characteristics of the study-communities and details on the scale validation are presented in the Additional files 1 and 2.

### Association between urbanicity and malaria

**Fig. 2:**
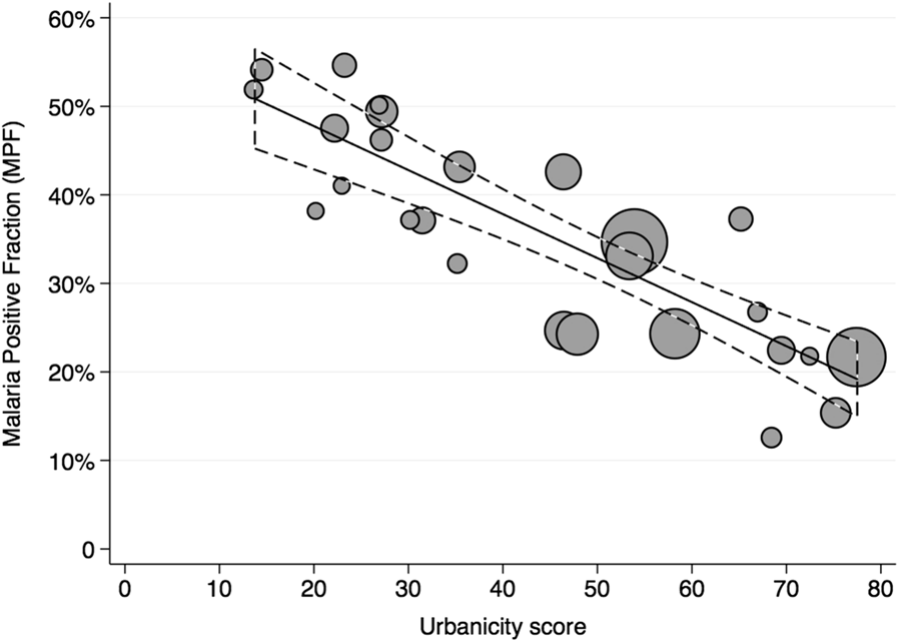
Association between urbanicity and MPF. The Figure illustrates a linear decrease of MPF with increasing levels of urbanicity. The linear regression models a 5 % decrease per ten-point increase on the urbanicity scale (95 % CI: 4–6 %, R^2^ = 0.70, p < 0.001). Data points are scaled proportional to the number of individuals contributing to the measurement of MPF

Overall, the MPF varied strongly between the communities with the highest MPF of 54.5 % in Aduaden, a small rural community in Bosumtwe District scoring 23.25 points on the urbanicity scale, and the lowest MPF of 12.5 % in Kaase, a suburb of Kumasi with 68.5 points on the urbanicity scale. Increasing urbanicity was strongly associated with a linear decrease in the MPF (R^2^ = 0.70) in the univariate linear regression model. For every ten points increase on the urbanicity scale the MPF decreased by 5 % (95 % confidence interval [95 % CI]: 4–6 %, p < 0.001), corresponding to a modelled MPF-decrease from 52 to 20 % over the measured range of the urbanicity scale (Table 3; Fig. 2).

**Table 3:** Linear regression models for associations between urbanicity and MPF, urbanicity and mean age of malaria patients, and MPF and mean age of malaria patients

Univariate regression models^a^				Multivariate regression models^a, b^			
Coefficient	95 % CI	p	Adj.-R^2c^	Coefficient	95 % CI	p	Adj.-R^2c^
Urbanicity and MPF^d^							
−4.97	−6.33–3.61	<0.001	0.69	−4.60	−6.03–3.17	<0.001	0.71
Urbanicity and age of malaria patients^e^							
2.93	1.04–4.81	0.004	0.27	2.33	0.24–4.44	0.03	0.29
MPF and age of malaria patients^f^							
−4.91	−8.11–1.73	0.004	0.27	−3.87	−7.50–0.25	0.04	0.28

^a^All models are weighted for the number of individuals contributing to the estimation of the outcome variables (MPF and mean age)

^b^Adjusted for ITN use

^c^Adjusted-R^2^

^d^Decrease in MPF (%) per ten-point decrease in urbanicity score

^e^Increase in mean age in months of patients with malaria per ten-point increase in urbanicity score

^f^Decrease in mean age in months of patients with malaria per 10 % increase in MPF

The use of ITNs was high in the study group with a proportion of 82 % (1769 of 2203 visits) of the mothers stating that their child had slept under an ITN the night before the hospital visit. This proportion varied from 68 % in Esereso to 100 % in Oyoko and there was no evidence for an association with the level of urbanicity (1 % decrease in ITN use per 10 % increase in urbanicity score, 95 % CI: −3–0.1 %, p = 0.07). Higher use of ITNs in the study group was associated with an increase in MPF [6 % increase in MPF per 10 % increase in ITN use (95 % CI: 1–11 %, p = 0.02)].

In the multivariate model for association between MPF and urbanicity, adjusted for ITN use, the explained variance hardly changed (R^2^ = 0.70) and the association between urbanicity and MPF was only slightly lower with a 4.6 % (95 % CI: 3–6 %, p < 0.001) decrease in MPF for each ten-point increase in urbanicity (Table 3).

### Malaria, urbanicity and acquisition of immunity

**Fig. 3:**
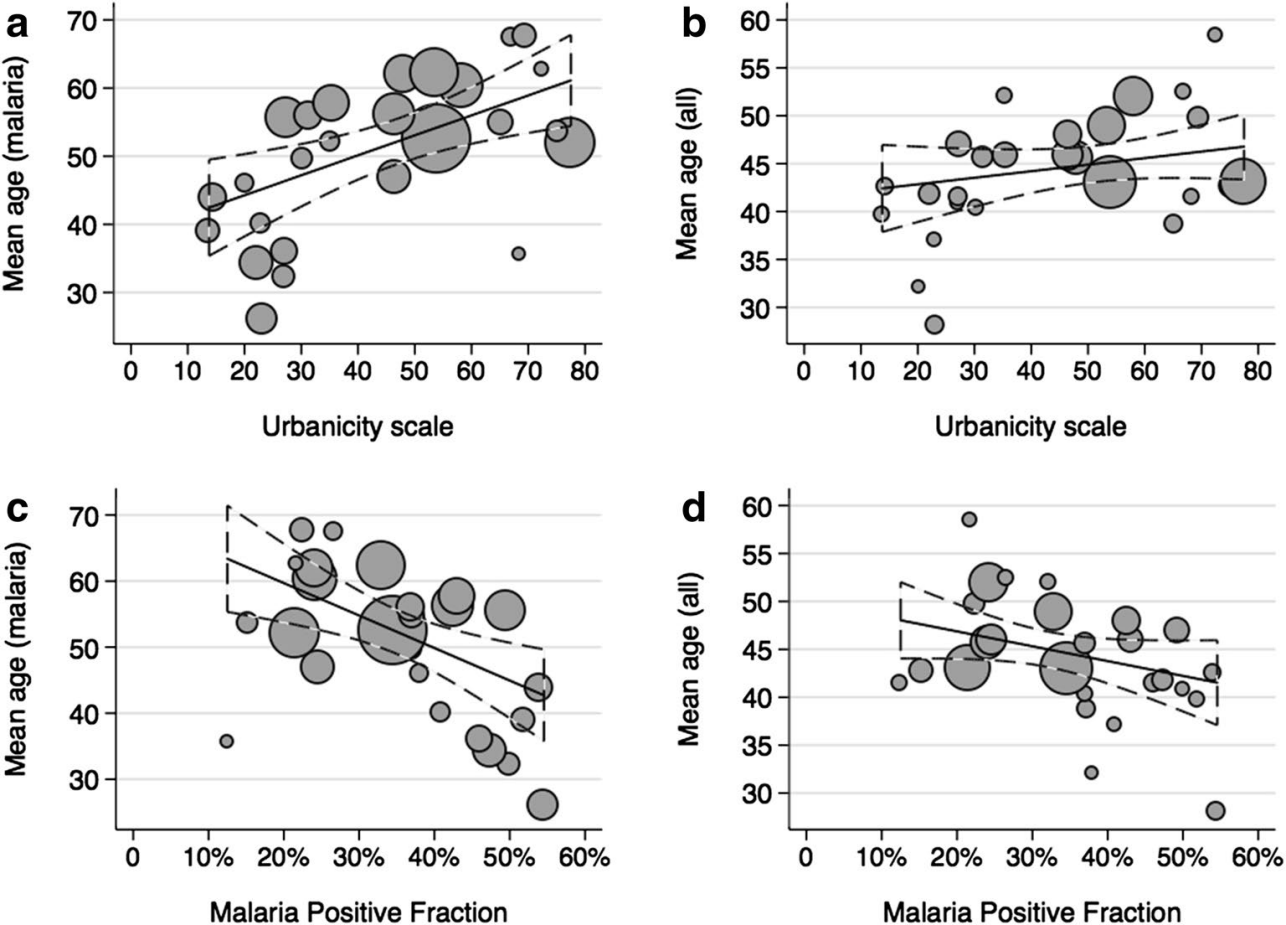
Association between urbanicity and age, and MPF and age. The Figure illustrates an association between urbanicity and the mean age of malaria patients in months (**a** R^2^ 0.27; p = 0.004), while there is no association between urbanicity and mean age of all patients (**b** R^2^ = 0.03; p = 0.21). In addition, there is an association between MPF and mean age of malaria patients (**c** R^2^ = 0.27; p = 0.004) and no association between MPF and mean age of all patients (**d** R^2^ = 0.08; p = 0.09). Data points are scaled proportional to the number of individuals contributing to the measurement of mean age

The mean age at presentation for patients with malaria varied strongly between communities, from 26 months in Aduadaden (23.25 points on the urbanicity scale) in Bosumtwe District to 68 months in Chirapatre (69.5 points on the urbanicity scale), a suburb of Kumasi. There was strong evidence for an association between urbanicity and mean age of malaria patients (R^2^ 0.27; p = 0.004) with an increase of 2.9 months (95 % CI: 1.0–4.8) per ten-point increase on the urbanicity scale (Table 3; Fig. 3a).

There was also strong evidence for a linear association between the mean age of malaria patients and community MPF (R^2^ 0.27; p = 0.004) with a modelled decrease of 4.9 months (95 % CI: 1.7–8.1) in the mean age per 10 % increase in MPF (Table 3; Fig. 3c). ITN use was also associated with mean age of malaria patients with a modelled decrease in mean age of 5.3 months per 10 % increase in ITN use (95 % CI: 0.8–9.7, p = 0.02). Adjusting the estimates for ITN use in multivariate models reduced both the association between mean age of malaria patients and urbanicity (2.3-month increase per ten points, 95 % CI: 0.2–4.4, p = 0.03) and mean age and MPF (3.9-month decrease per 10 %, 95 % CI: 0.2–7.4, p = 0.04).

The mean age at presentation for all patients also varied strongly between communities, from 28 months in Aduadaden (23.25 points on the urbanicity scale) in Bosumtwe District to 58 months in Gyinyase (72.5 points on the urbanicity scale), a suburb of Kumasi. However, there was no association with urbanicity (*β* = 0.007; R^2^ = 0.03; p = 0.21, Fig. 3b), or MPF (*β* = −1.1; R^2^ = 0.08; p = 0.09, Fig. 3d).

### Single score-components and MPF

The bivariate models showed that all components correlate negatively with MPF (Table 4), while the strongest associations were observed for population size (coefficient −0.04, R^2^ 0.59), economic activity (coefficient −0.04, R^2^ 0.57), education (coefficient −0.04, R^2^ 0.52), and housing (coefficient −0.04, R^2^ 0.65). In the multivariate model no variable remained associated with MPF, but R^2^ squared increased to 0.77. The model showed the strong co-linearity between components, which was also seen in the exploratory factor analysis performed to validate the score (see Additional file 1).

**Table 4:** Linear regression models for associations between components of the urbanicity score and MPF

Variable^a^	Univariate regression			Multivariate regression		
	Coefficient (95 % CI)	*p* value	Adjusted-R^2^	Coefficient (95 % CI)	*p* value	Adjusted-R^2^
Population size	−0.04 (−0.06 to 0.03)	<0.001	0.59	−0.01 (−0.03 to 0.02)	0.67	0.77
Economic activity	−0.04 (−0.05 to −0.02)	<0.001	0.57	−0.01 (−0.04 to 0.01)	0.29	
Education	−0.04 (−0.06 to −0.24)	<0.001	0.52	−0.02 (−0.05 to 0.01)	0.16	
Health services	−0.02 (−0.03 to −0.01)	<0.001	0.40	0.01 (−0.01 to 0.02)	0.80	
Transportation	−0.02 (−0.04 to −0.01)	0.001	0.40	−0.01 (−0.02 to 0.01)	0.32	
Services	−0.02 (−0.03 to −0.01)	<0.001	0.54	−0.01 (−0.01 to 0.03)	0.84	
Sanitation	−0.02 (−0.03 to −0.02)	<0.001	0.54	0.01 (−0.01 to 0.02)	0.40	
Housing	−0.04 (−0.06 to −0.03)	<0.001	0.65	−0.02 (−0.06 to 0.02)	0.32	

^a^Each component ranges from 0 to 10 points

## Discussion

Using data from a hospital-based study, an ecological analysis of the association between urbanicity and clinical malaria episodes was performed. Using MPF as outcome and a continuous measure of urbanicity as exposure, a linear decrease of 5 % in MPF with every ten-point increase in urbanicity was identified. In addition, it was found that the mean age of malaria patients increased by 2.9 months with every ten-point increase on the urbanicity scale, indicating a potential influence of urbanicity on the acquisition of semi-immunity against malaria. This is supported by the finding of a lower mean age of malaria patients in communities with a higher MPF.

Through the use of a continuous scale of urbanicity that provides more detailed information than a binary classification, it was possible to assess linear associations with the covariates. The vast majority of previous studies [3, 5, 8, 9, 20, 21] used a crude rural–urban dichotomy that is more arbitrary and cannot distinguish localities and cities on a continuum of urbanicity. In contrast to these studies, this continuous scale is based on standardized methods of scale construction [17] that have been applied and validated to measure urbanicity in different settings [22–24].

The results confirm heterogeneity of malaria prevalence in areas of high endemicity as previously reported [10] and underpin the postulated association between urbanization and the decreasing incidence of malaria. The most detailed study on the effect of urbanization on malaria was conducted in the Republic of Congo between 1982 and 1984 and found high spatial variation of malaria prevalence in Brazzaville [8, 25, 26]. Water bodies in urban areas were scarce or polluted and thus unfavourable as breeding sites for mosquitoes. The authors also found that the flight range of mosquitoes was reduced from several kilometres in rural areas to a few 100 m in urban areas [8], probably due to higher human population density. Data from the city of Dakar showed highly focused malaria transmission and a decreasing malaria risk in urbanized areas [27, 28]. More recently, a study from Ouagadougou showed a decreasing risk of malaria from the periphery of the city towards the urban centre and identified urban agriculture and residence near mosquito breeding sites as risk factors for urban malaria [20]. The relationship between urbanicity and malaria transmission has been modelled on a global scale in several studies. These models found a pattern of decreasing malaria transmission coinciding with increased urbanization [3, 5, 9]. Furthermore, in a meta-analysis of entomological inoculation rate (EIR) surveys, lower EIRs in urban or peri-urban areas compared to rural areas were found [21]. The decline in transmission in urban areas is best explained by a rise in socio-economic status, housing quality and access to infrastructure, sanitation and education, as well as a higher use of prevention methods (e.g., ITNs) and access to healthcare [29, 30]. In the study presented here, the strongest associations between MPF and single components of the score were observed for population size, economic activity, education, and housing. However, the observed unidimensionality of the score components supports the approach to combine the components into one measure of urbanicity rather than assessing component’s associations with MPF.

The use of ITNs in the current study was not significantly associated with urbanicity, but surprisingly associated with a higher MPF. However, the analysis was performed on an ecological level and individual risk was not assessed. Therefore, ITN use may have been higher in communities with a high MPF as a response to higher transmission there. The reported use of ITNs was also higher than in previous studies where 22 % of children below the age of five slept under a bed net in 2006 [31]. This difference is possibly due to a recent upscaling in ITN distribution [32] or a result of a possible interviewer bias.

The findings of varying mean ages of malaria patients over short distances and a correlation of mean age with urbanicity and MPF indicate that the acquisition of immunity is spatially heterogeneous and possibly slower in urban areas. The association between prevalence of malaria and the acquisition of immunity is well known and has been studied on a macro-epidemiological scale and in modelling approaches [12, 33, 34]. A recent meta-analysis found that clinical malaria is experienced at all ages, but the age of severe symptoms is inversely associated with transmission intensity [35]. However, data on heterogeneity in immunity on a micro-epidemiological level are scarce and there are few studies on the effect of urbanicity. An early, cross-sectional study from southern Ghana found a markedly lower prevalence of antibodies against *P. falciparum* in an urban setting compared to a rural area [36]. However, antibody responses to malaria are not necessarily correlated to immunity and may just be markers of a higher exposure in rural areas. Several studies have looked at the mean age of malaria patients in regions or countries of low and high transmission intensity [37, 38] and as a change over time with decreasing malaria incidence [39]. All these studies consistently found a lower mean age of malaria in higher transmission settings. However, to the authors’ knowledge the only study that looked at varying age of infection over short distances is a recent ecological analysis from Kenya that also found a correlation between MPF and mean age of infection [40].

A difference in the age of infection between urban and rural areas and a possible delay in the acquisition of immunity has several implications for clinicians and intervention programmes. First, severe disease in urban areas may be more evenly distributed across childhood [41] and clinicians should also consider malaria as a cause of severe febrile illness in older children. Second, intervention programmes focusing on infants may not be accurately targeted in the future even if the brunt of malaria deaths is still borne by the youngest children today [35]. Third, delayed and potentially waning immunity may lead to populations susceptible to severe disease and potentially to epidemics of severe malaria.

While these findings could have major implications, the data should be interpreted with care. First, MPF is a measure of disease prevalence in patients attending the hospital and not a direct measure in the community. Calculating malaria prevalence/incidence based on data from healthcare facilities is biased by access to care and unknown hospital coverage. By using the MPF among children with fever, as previously done in several studies [40, 42], the measure of prevalence is not biased by access to care. MPF, as a measure within the population attending the hospital, would only be influenced by healthcare-seeking behaviour if it is different between malaria cases and fever cases with other causes (e.g., if mothers took children with malaria to private clinics and attended the hospital with other infections). As malaria is clinically indistinguishable from other febrile infections, such an effect is unlikely to have biased the results. However, it is impossible to know whether the families attending the hospital are representative of the communities that they come from and the findings may therefore not be transferable. Second, MPF was calculated based on all cases of malaria presenting to an OPD and did not stratify for degrees of disease severity. The pattern of association between MPF and urbanicity might vary by disease severity [35] and further studies are needed to study this in detail. Third, malaria infection may have been acquired while travelling outside residential areas and therefore falsely attributed to the community of residence in this analysis. However, this limitation is less likely for children and the main vector in the area, *Anopheles gambiae* is known to be mainly endophilic [43]. Fourth, as immunity is influenced by changes in urbanicity, which is an ongoing process, the population immunity may not be at equilibrium with the environment. However, as the data presented here are from children with a mean age of 3.3 years, changes in urbanicity over such a short period are unlikely to be large enough to have a strong effect on the effects seen here. Finally, this is a cross-sectional study and the data were analysed on an ecological level, making causal inferences difficult.

The results demonstrate the feasibility of hospital-based studies to analyse crude associations in the micro-epidemiology of malaria and acquired immunity. However, further studies including clinical data on disease severity, especially cerebral malaria, and analyses on an individual level are needed. Future studies should also include additional risk factors such as surrounding breeding sites. Such studies will lead to a better understanding of the heterogeneity of both transmission and immunity and provide data for geographically targeted interventions.

## Additional files

10.1186/s12936-016-1138-4 Description of urbanicity score.
10.1186/s12936-016-1138-4 Table containing characteristics per community.
