# Supplementary material for: Spatial heterogeneity of malaria in Ghana: a cross-sectional study on the association between urbanicity and the acquisition of immunity
Source: Malar J. 2016 Feb 11;15:84. doi: 10.1186/s12936-016-1138-4 (PMC4751679; doi:10.1186/s12936-016-1138-4)
Supplement: Supplementary file 1 — 10.1186/s12936-016-1138-4 Description of urbanicity score. [file 12936_2016_1138_MOESM1_ESM.docx]

**Supplementary Material:**

**Methods of urbanicity** **scale construction**

*Variable selection and set-up of components*

The scale was constructed and validated according to the principles of scale development as described by DeVellis [1] and Netemeyer & Bearden [2].

Based on the current literature [3–7], several variables known to be associated with urbanicity were selected. These variables were grouped into eight logical components. Components used to set up the score were: Population size, economic activity, education, health services, transportation, services, sanitation, and housing. The variables included in each component are presented in Table 1.

*Scoring for each variable*

To transform the village characteristics into a numerical scale, each of the eight components received a maximum of 10 points, resulting in a theoretical scale range from 0 to 80 points. Within each component, between one and five variables were grouped, depending on data availability. Each variable contributed equally to the components maximum score. Thus, the maximum points (i.e., 10) were divided by the number of variables grouped within each component. Finally, variables scored between 2 and 10 points (Table 1, main manuscript).

To calculate the score for a community score points were summarised based on the absence or presence of particular urbanicity feature. Categories of dichotomous variables were assigned 0 points or the maximum points assigned to the specific variable (e.g., availability of a primary school in a community: 2 points; absence of a primary school: 0 points). For continuous variables each variable’s range and distribution were assessed and a reasonable number of quantiles was established. Accordingly a locality scored zero whenever it was ranked in the lowest quantile of a variable and it scored maximum whenever it was ranked in a highest quantile. Data on village characteristics used to construct the score were retrieved from the Ghana Statistical Service [8] or were collected by the study team.

*Validation*

Validation was performed as described by DeVellis [1]. Unidimensionality was tested using exploratory factor analysis (EFA) [2]. The Kaiser-Meyer-Olkin measure of sampling adequacy was performed to assess whether variable correlation can be calculated via factor analysis [2]. Internal consistency was validated by Cronbach’s alpha as a measure for shared covariance [7]. In addition, each component’s correlation to the remaining components was examined by calculating corrected item-scale correlations [1].

As there is no gold-standard to assess urbanicity, criterion related validity was assessed by comparing the score to the dichotomous classification by the Ghanaian Statistical Service, which uses only population size as a measure for urbanicity (cut-off: 5000). The score was dichotomized using a receiver-operator curve with maximized sensitivity and specificity. Both classifications were compared using a Kappa statistic to assess the agreement beyond chance.

**Results**

The scale was constructed for 73 communities, while 26 communities were included for the analysis. This explains that some values may differ from the main article. The scale ranged from 6 to 78.5 points, with a mean of 40.2 (SD: 23.9) and a median of 31.25 (IQR: 20.5 – 65.25) points. The exploratory factor analysis showed an eigenvalue of 6.06 for the first factor, explaining 96.6% of the overall variance. All subsequent factors showed an eigenvalue <1. The Kaiser-Meyer-Olkin measure showed an overall value of 0.93 for the first factor, which indicated that components share correlation pattern to be expressed by just one factor [9]. Cronbach’s alpha showed a high internal consistency of the scale (α =0.96), average shared inter-item correlation was 0.75 and the corrected item-scale correlations ranged from 0.68 to 0.92. Sensitivity and specificity of the dichotomized score were 92% and 96%, respectively and 94.5% of all communities were classified correctly. The comparison with the dichotomous classification used by the Ghana Statistical Service revealed a kappa-value of 0.88 indicating almost perfect agreement between both classifications [10].

**References**

1. DeVellis RF: *Scale Development: Theory and Aplications*. Second Edi. *Volume 26*. Tousand Oaks: Sage Publications Inc.; 2003.
2. Netemeyer RG, Bearden WO, Sharma S: *Scaling Procedures - Issues and Applications*. Tousand Oaks: Sage Publications Inc.; 2003.
3. McDade TW, Adair LS: **Defining the “urban” in urbanization and health: a factor analysis approach.** *Soc Sci Med* 2001, **53**:55–70.
4. 4. Dahly DL, Adair LS: **Quantifying the urban environment: a scale measure of urbanicity outperforms the urban-rural dichotomy**. *Soc Sci Med* 2007, **64**:1407–1419.
5. Van de Poel E, O’Donnell O, Van Doorslaer E: **Urbanization and the spread of diseases of affluence in China.** *Econ Hum Biol* 2009, **7**:200–16.
6. Novak NL, Allender S, Scarborough P, West D: **The development and validation of an urbanicity scale in a multi-country study.** *BMC Public Health* 2012, **12**:530.
7. Jones-Smith JC, Popkin BM: **Understanding community context and adult health changes in China: development of an urbanicity scale.** *Soc Sci Med* 2010, **71**:1436–1446.
8. Ghana Statistical Service: *GSS Population & Housing Census*. 2010.
9. Kaiser HF: **An index of factorial simplicity**. *Psychometrika* 1974, **39**:31–36.
10. Landis JR, Koch GG: **The measurement of observer agreement for categorical data.** *Biometrics* 1977, **33**:159–74.
