# Supplementary material for: Spatial heterogeneity of malaria in Ghana: a cross-sectional study on the association between urbanicity and the acquisition of immunity
Source: Malar J. 2016 Feb 11;15:84. doi: 10.1186/s12936-016-1138-4 (PMC4751679; doi:10.1186/s12936-016-1138-4)
Supplement: Supplementary file 2 — 10.1186/s12936-016-1138-4 Table containing characteristics per community. [file 12936_2016_1138_MOESM2_ESM.doc]

| **Table 1:** Score points on the urbanicity scale for the villages included into the analysis. | | | | | | | | | | |
| --- | --- | --- | --- | --- | --- | --- | --- | --- | --- | --- |
| **Villages** | **Inhabitants** | **Urbanicity Score** | **Population Score** | **Economic Score** | **Education Score** | **Health Score** | **Transport Score** | **Service Score** | **Sanitation Score** | **Housing Score** |
| Abijan nkwanta (Bosomtwe) | 1587 | **46.5** | 5 | 7 | 5 | 0 | 10 | 3.75 | 8.75 | 7 |
| Abonu (Bosomtwe) | 1007 | **30.25** | 3 | 5 | 5.5 | 0 | 10 | 1.25 | 2.5 | 3 |
| Abuontem (Bosomtwe) | 3132 | **35.5** | 6 | 6 | 7 | 0 | 5 | 1.25 | 6.25 | 4 |
| Adagya (Bosomtwe) | 3261 | **31.5** | 6 | 6 | 3.5 | 0 | 5 | 1.25 | 3.75 | 6 |
| Aduaden (Bosomtwe) | 1665 | **23.25** | 5 | 3 | 4 | 0 | 5 | 1.25 | 2.5 | 2.5 |
| Adwafo (Bosomtwe) | 1005 | **20.25** | 3 | 1 | 5 | 0 | 5 | 0 | 3.75 | 2.5 |
| Ahinsan (Kumasi) | 27264 | **75.25** | 10 | 9 | 8.5 | 10 | 10 | 10 | 8.75 | 9 |
| Akokofe (Bosomtwe) | 663 | **23** | 2 | 3 | 5 | 0 | 10 | 0 | 0 | 3 |
| Aputuogya (Bosomtwe) | 5414 | **48** | 7 | 7 | 5 | 0 | 10 | 6.25 | 6.25 | 6.5 |
| Atonsu-Agogo (Kumasi) | 65225 | **77.5** | 10 | 9 | 9.5 | 10 | 10 | 10 | 10 | 9 |
| Chirapatre (Kumasi) | 15815 | **69.5** | 9 | 8 | 10 | 10 | 10 | 5 | 10 | 7.5 |
| Dedesua (Bosomtwe) | 650 | **13.75** | 2 | 5 | 2.5 | 0 | 0 | 1.25 | 0 | 3 |
| Dompoase (Kumasi) | 6966 | **67** | 7 | 8 | 10 | 10 | 10 | 5 | 10 | 7 |
| Esereso (Bosomtwe) | 8150 | **58.25** | 8 | 9 | 7.5 | 5 | 10 | 5 | 6.25 | 7.5 |
| Feyiase (Bosomtwe) | 7474 | **46.5** | 7 | 7 | 7 | 0 | 10 | 3.75 | 6.25 | 5.5 |
| Gyakyi (Bosomtwe) | 7337 | **54** | 7 | 5 | 8 | 5 | 10 | 7.5 | 5 | 6.5 |
| Gyinyase (Kumasi) | 14298 | **72.5** | 8 | 7 | 10 | 10 | 10 | 10 | 10 | 7.5 |
| Kaase (Kumasi) | 15697 | **68.5** | 8 | 8 | 8.5 | 10 | 10 | 8.75 | 8.75 | 6.5 |
| Kokodei (Bosomtwe) | 882 | **14.5** | 3 | 0 | 5.5 | 0 | 5 | 0 | 0 | 1 |
| Kuntanase (Bosomtwe) | 3583 | **65.25** | 6 | 6 | 8.5 | 10 | 10 | 10 | 8.75 | 6 |
| Nnuaso (Bosomtwe) | 919 | **22.25** | 3 | 4 | 6 | 0 | 5 | 0 | 1.25 | 3 |
| Nyamiani (Bosomtwe) | 1608 | **27.25** | 5 | 2 | 5.5 | 5 | 5 | 0 | 1.25 | 3.5 |
| Oyoko (Bosomtwe) | 1288 | **27** | 4 | 3 | 5.5 | 0 | 5 | 0 | 5 | 4.5 |
| Pramso (Bosomtwe) | 3259 | **53.5** | 6 | 5 | 8 | 10 | 10 | 5 | 5 | 4.5 |
| Sawua (Bosomtwe) | 3119 | **35.25** | 6 | 6 | 6.5 | 0 | 5 | 1.25 | 5 | 5.5 |
| Soaduro (Bosomtwe) | 1580 | **27.25** | 5 | 4 | 4 | 0 | 10 | 0 | 1.25 | 3 |
